# Alternative Strategies for Multi-Stress Tolerance and Yield Improvement in Millets

**DOI:** 10.3390/genes12050739

**Published:** 2021-05-14

**Authors:** Muhammad Numan, Desalegn D. Serba, Ayalew Ligaba-Osena

**Affiliations:** 1 Laboratory of Biotechnology and Molecular Biology, Department of Biology, University of North Carolina at Greensboro, 321 McIver Street, Greensboro, NC 27412, USA; m_numan@uncg.edu; 2 USDA-ARS, U. S. Arid-Land Agricultural Research Center, 21881 N Cardon Ln., Maricopa, AZ 85138, USA; des.serba@usda.gov

**Keywords:** millets, abiotic stress, genome editing, rhizobacteria, CRISPR/Cas9

## Abstract

Millets are important cereal crops cultivated in arid and semiarid regions of the world, particularly Africa and southeast Asia. Climate change has triggered multiple abiotic stresses in plants that are the main causes of crop loss worldwide, reducing average yield for most crops by more than 50%. Although millets are tolerant to most abiotic stresses including drought and high temperatures, further improvement is needed to make them more resilient to unprecedented effects of climate change and associated environmental stresses. Incorporation of stress tolerance traits in millets will improve their productivity in marginal environments and will help in overcoming future food shortage due to climate change. Recently, approaches such as application of plant growth-promoting rhizobacteria (PGPRs) have been used to improve growth and development, as well as stress tolerance of crops. Moreover, with the advance of next-generation sequencing technology, genome editing, using the clustered regularly interspaced short palindromic repeats (CRISPR/Cas9) system are increasingly used to develop stress tolerant varieties in different crops. In this paper, the innate ability of millets to tolerate abiotic stresses and alternative approaches to boost stress resistance were thoroughly reviewed. Moreover, several stress-resistant genes were identified in related monocots such as rice (*Oryza sativa*), wheat (*Triticum aestivum*), and maize (*Zea mays*), and other related species for which orthologs in millets could be manipulated by CRISPR/Cas9 and related genome-editing techniques to improve stress resilience and productivity. These cutting-edge alternative strategies are expected to bring this group of orphan crops at the forefront of scientific research for their potential contribution to global food security.

## 1. Introduction

Millets are considered as major cereal crops in the developing world. In the semiarid tropical areas of Asia and Africa, they are especially important because of their use as human food, as well as feed for livestock [1,2,3]. Millets are cultivated in marginal environments and represents small grain crops [4,5,6]. Finger millet (*Eleusine coracana* (L.) Gaertn), pearl millet (*Pennisetum glaucum* (L.) R. Br), kodo millet (*Paspalum scrobiculatum* L.), Japanese barnyard millet (*Echinochloa esculneta*), proso millet (*Panicum miliaceum* L.), foxtail millet (*Setaria italica* (L.) P. Beauvois), little millet (*Panicum sumatrense* Roth ex Roem. & Schult.), tef (*Eragrostis tef* (Zucc.) Trotter), and Indian barnyard millet (*Echinochloa frumentacea* Link) are traditionally considered as millets [7,8,9]. Pearl millet is an exception to small grain crops due to its morphological features [10,11].

One of the major attributes of millets is their high nutritional value as compared to other cereal crops [12,13,14]. The protein profile of millets shows that they contain higher amounts of methionine and other essential amino acids [15,16]. Few studies also reported that millets are rich in micronutrients and phytochemicals [17,18,19] that have health benefits. For example, antioxidant enzymes, insoluble and soluble dietary fibers, and resistant starch are abundantly found in pearl millet [15,17,18,19]. Biochemical profiling revealed that pearl millet contains 63% starch, 13% protein, 7% fats, 2% crude fibers, and about 92% dry matter [7,15,16]. Foxtail millet is used as a supplementary source of protein for other cereals because of the presence of the essential amino acid lysine [15,17,18,19]. Finger millet is rich in polyphenols and other essential phytochemicals [15,17,18,19,20], and it has a high amount of calcium, methionine, tryptophan, fiber, and sulfur-containing amino acids [21]. Finger millet contains minerals (2%), crude fibers (4%), protein (9%), and carbohydrate (81%) [22,23]. The mineral and fiber contents of finger millet are higher than those of wheat and rice. Finger millet also contains more valine, threonine, and lysine than other millet species [15,17,18,19,22]. The presence of essential nutrients and phytochemicals with health benefits make millets great resources for utilization in food industries [7,15,17,18,19,22].

Despite the great benefits of millets for humans, their yield is limited by multi-environmental stresses and the looming climate change [24,25]. Combined heat and drought stress has posed a serious threat to the productivity of these crops [26,27]. Drought stress at the seedling stage [28] and terminal drought during the reproductive stage have been shown to incur up to 60% and 40% of yield loss in pearl millet and tef, respectively [28,29,30]. In Africa, particularly in the sub-Saharan countries, about 40% of the population lives in a drier environment; however, this number is increasing and is expected to double by 2050 [31]. Drought accompanied by heat stress has a considerable effect on the physiological, cellular, and molecular functions of plants. Photosynthesis and respiration are among the major processes affected by these stresses, which determine crop yield and productivity [26,27,32]. Studies suggested that a temperature above the threshold level (28 °C to 32 °C) has very harmful effects, for example, by limiting the adaptation potential of a crop [26,27,32,33]. An increase in temperature of about 3–4 °C can reduce crop yields by up to 35% [32,34]. Reports on global warming have suggested that south Asia and Africa are regions most affected by climate change, primarily global warming [35,36]. It was reported that the average annual increase in temperature since 1980 has been 0.17 °C (0.31 °F) [32,34,37]. A direct link between decreased crops yields and combined drought and heat stress has also been reported [26,27,32,38].

In order to overcome the effects of climate change-associated stresses and to improve the yield of millets, there is a need to develop stress-tolerant and high-yielding varieties. There are several possible approaches to increase the stress tolerance and productivity of millets. For example, application of PGPRs has been used to improve yield and stress tolerance in wheat [39,40,41] and rice [42,43,44], but it remains to be tested in millets. Another approach for trait improvement is via genome editing, which has recently gained tremendous attentions due to its specific allele manipulation potential. The CRISPR/Cas9 system has emerged as a promising technique to edit plant genome/genes for stress tolerance and higher productivity. CRISPRs, along with their related proteins known as Cas, are widespread among the phyla of archaea and bacteria where they function as an adaptive immune system against phages [45]. The CRISPR loci acquire short sequences of their DNA as spacers and, thus, carry information regarding previous plasmid infections or bacteriophages upon recognition of invading DNA [46]. 

Conventional breeding approaches such as selection from landraces, hybridization to create new variability followed by pedigree selection, mutation breeding, and exploitation of hybrid vigor have resulted in significant improvement and release of new cultivars in different millets [47,48,49]. However, the yields of millets remain much lower than major cereals even under optimum growth conditions. Following the advent of next-generation sequencing, markers and genomics-assisted breeding approaches have been implemented to improve these group of crops. The genome sequences of foxtail millet [50], tef [51,52], pearl millet [53], finger millet [21], and proso millet (broomcorn) [54] have become available and are very useful resources for genetic improvement of these crops. 

Although millets have recently gained attention for their various food and health benefits, as well adaptation to adverse environmental conditions, their productivity has remained low, and which needs to be boosted to enhance their utilization as a food crop globally. This review article highlights the combined effects of drought and heat on millets concurrent with looming climate change, as well as their inherent genetic potential as climate smart crops, and proposes potential alternative strategies for the improvement of stress tolerance and yield of these important group of crops.

## 2. Effects of Climate Change on Millets and Their Tolerance Strategies

Climate change and its impact on agriculture crops has been widely reviewed [55,56,57,58,59,60]. The climate is changing at an alarming rate, triggering various abiotic stresses which are affecting food crops in one way or another, which directly affects the global food supply. The major effects of climate change on millet crops include drought stress, heat stress, flooding or waterlogging stress, and lodging. 

### 2.1. Effects of Drought Stress

The arid and semiarid lands of most developing countries are facing a problem of water scarcity, which affects the type and performance of crops grown in those areas. A study performed on wild millet (*Setaria glauca*), foxtail millet (*Setaria italica*), little millet (*Panicum sumatrense*), and proso millet (*Panicum miliaceum*) reported a significant reduction in yield when subjected to drought prior to flowering [61]. Likewise, complete yield loss was reported in two finger millet landraces subjected to drought after 4 weeks of sowing [61,62]. About 60% yield loss was recorded due to terminal drought that occurred during flowering through maturity [63]. The estimated yield loss due to drought in pearl millet was about 51% [64,65], and, in tef, it was estimated to be 40% [66,67], as shown in Table 1. Millets including tef can generally survive at very low soil moisture content. For example, it was reported that, in the Sahel region where the moisture level is extremely low, the biomass of millet and sorghum was comparable [68]. Undesirable effects of drought have been reported not only on the productivity of crops but also on their nutritional quality including grain mineral and protein content [12,61,69,70].

Plants employ various abiotic stress tolerance mechanisms [71,72] to thrive in drought-prone regions. Four major adaptation mechanisms of millets to drought-inflicted regions of the world were recently reviewed [73]. These mechanisms include (i) drought avoidance, which is the capability of plant to sustain the balance of water during stress to avoid water deficiency in tissues, (ii) drought tolerance, referring to plants’ ability to produce biomass by withstanding reduced water potential, (iii) drought escape, a state in which plants mature prior to drought stress, and (iv) drought recovery, a condition in which plants provide some yield by recovering from intermittent drought effects after moisture becomes available.

In sub-Saharan African, millets are adapted to low water potential, and grown for sustenance. These crops are presumed to guarantee food security in the future as they can be used as model for stress tolerance in crop improvement or as an alternative crop in drought-inflicted areas [74]. Drought tolerance strategies in millets also involve physiological modifications including stomatal conductance, osmotic adjustment, and cell membrane stability [75]. Among these, osmotic adjustment enables the plant leaves to maintain leaf turgor pressure (LTP) [76], even under extreme drought conditions, by retrieving and absorbing water even from dry soils [76,77]. Increased root elongation is another drought stress tolerance mechanism [78,79]. For example, the increase in root length of legumes such as cowpea (*Vigna unguiculata*), peanut (*Arachis hypogaea*), and soybean (*Glycine max*), when exposed to drought enables them to absorb water at greater soil depth [79]. Similarly, Ayele, et al. [80] and Debieu, et al. [81] reported that a deeper, more extensive, and broader root system in tef was shown to provide drought stress tolerance.

### 2.2. Effects of Heat Stress

Although most millet species are resistant to heat stress, heat induces many physiological and molecular alterations. Photosynthesis and respiration are the most sensitive processes to heat stress, which has a dramatic effect on crop productivity [82]. Significant yield loss due to heat has already been reported in many crops [83], and the annual increase in temperature due to climate change poses a threat to food security. Crop yield increases with increasing temperatures up to a threshold level (cotton 32 °C, soybean 30 °C, and maize 29 °C); however, above the threshold level, a slight increase in temperature has severe negative effects on plant growth and ultimately yield [84]. Yield loss of up to 35% has been reported due to a 3–4 °C increase in temperature (Table 1). As estimated by statistical modeling, climate change and global warming are likely to severely affect cereal production in Africa and Asia [85]. 

It has been established that high-temperature stress reduces transport of electrons, disrupts the function of photosystem (PS) II, and enhances the amount of ROS accumulation [86]. It also desiccates the reproductive parts and can result in plant sterility, seed abortion, reduced seed number, and shortened grain filling period [87]. Over time, through evolution, plants have developed various acclimation, avoidance, and adaptive strategies to deal with heat stress. Mechanisms of tolerance involve upregulation of the antioxidant system, transcription factors, heat-shock proteins, signaling molecules, ion transporters, and accumulation of osmoprotectants [88]. Plant membranes are prone to lipid peroxidation, and, in wheat, membrane thermal stability has been used as a selection criterion for heat tolerance [89]. Under normal conditions, cell metabolism produces ROS as a byproduct, which at high concentration can induce oxidative stress. Most plants use pathways containing antioxidants to combat ROS as protective gear against various abiotic stresses [90]. Acquired heat tolerance has been observed in cowpea [91], chickpea (*Cicer arietinum*) [92], and pearl millet [93]. In chickpea, identification of 18 single-nucleotide polymorphisms (SNPs) from five stress-responsive genes has been reported, which include *dehydration responsive element binding (DREB)*, *abscisic acid-*, *stress-*, and *ripening-induced (ASR)*, *NAD^+^-dependent aminoaldehyde dehydrogenase (AMDH)*, *ERECTA* (*ER*), and *cyclase-associated proteins (CAP2) promoter*. These genes were highly associated with different adaptive traits under drought and heat stress [92].

### 2.3. The Effect of Waterlogging Stress on Millets

Waterlogging stress is the main cause of low productivity in high-precipitation areas [94]. Under waterlogging stress, the soil pores are filled with water that leads to the accumulation of toxic compounds, and inhibition of gas diffusion. This eventually affects roots, stomatal conductance, and photosynthesis [95]. Crops like wheat and maize are mostly affected by waterlogging in black clay soil (Vertisols), which has high water-holding capacity. Grain yield losses of about 18% in wild millet and 16% in proso millet were reported with waterlogging treatment that lasted from two weeks after planting through crop maturity [95], as shown in Table 1.

Plants have various mechanisms to cope with waterlogging stress [96], which are induced by hypoxia (reduced oxygen level) or anoxia (complete absence of oxygen). Plants respond to diminished oxygen by carrying out anaerobic respiration, which has also been reported in finger millet (*Eleusine coracana*) [97]. Anaerobic metabolism is not as efficient as aerobic metabolism, but ATP produced through fermentation supports the cell for a short period. This mechanism requires more sugar than aerobic metabolism; thus, alterations in carbohydrate metabolism are observed in waterlogging tolerant species such as finger millet and rice [98]. During waterlogging stress, spongy tissue containing air gaps, i.e., aerenchyma, which allow gases to move to roots from stems, are formed in tolerant plants [99]. These spaces are developed without (*schizogenous*) or with (*lysigenous*) cell death [100]. Studies showed that, in stressed sunflower (*Helianthus annuus*), aerenchyma (*lysigenous*) are developed within 2 days of the onset of stress [101]. Another strategy employed by waterlogging-tolerant species includes the formation of adventitious roots [96]. Development of adventitious roots has been observed in finger millet [102] and sorghum (*Sorghum bicolor*) [103]. Other crops such as mung beans (*Vigna radiata*) are known to escape logging through fast growth [104]. Additionally, waterlogging-tolerant plant possess abundant solubilized sugar [105], while plants such as tef respond to waterlogging stress by enhancing the activity of nitrogen reductase in the shoots [106].

### 2.4. Lodging Effects on Millet Yield

Lodging is the permanent bending of the stem from an upright position that is a common problem in millet crops. Forces such as irrigation water, rain, wind, or their combination can induce lodging. For example, lubrication of soil by rain water in combination with wind can push the plants toward the soil [107]. The stems and roots are two main targets of lodging stress, which are described in the literature as stem lodging and root lodging, respectively [108,109]. In stem lodging, the stems tilt toward the soil or break, whereas root lodging is associated with a change in the angle between the stem and soil due to the wind force on the stem or crown bending/root disanchoring [110]. Pinthus [107] reported that, before the stem breaking, more force is exerted to the plant part interacting with the soil. Contrarily, stem flexibility enhances the swing time of the stem, which increases the damage due to minor wind force, thus leading to lodging stress in most crops.

Several studies have reported the negative impacts of lodging stress on yields of tef and foxtail millet crops (Table 1). In tef, the leaf and stem architecture are very delicate, and the plant is susceptive to root lodging [111]. Lodging stress can also reduce yield in foxtail millet Tian, et al. [112]). Another study performed by Opole [113] showed excess fertilizer to cause lodging stress in finger millet, which ultimately reduces yield. Pearl millet is resistant to lodging, as well as various other biotic and abiotic stresses [114]. 

Generally, most millet crops are resistant to abiotic stresses including lodging under low-input conditions as compared to other cereal crops. However, new strategies need to be developed, or existing techniques need to be improved to enhance lodging tolerance in millet crops to benefit from fertilizer application to increase yields. Reducing plant height by genetic manipulation or exogenous application of chemicals is a common strategy to overcome lodging stress in cereals. For example, during the green revolution, the genes (Rht dwarfing genes) responsible for reducing plant height were introduced into wheat to enhance input responsiveness, e.g., to nitrogen fertilizer [115]. Other crop management practices that can reduce lodging are changing the seed sowing date, tilling practices, and increasing the intra-row space or reducing the number of plants in a row [107,109,110]. Moreover, application of silicon amendments has been shown to increase the yield of millets and other crops such as rice and sugar cane [116,117,118,119,120]. In rice, Si treatment was reported to strengthen the stem by increasing silica deposition in the shoot, increasing the thickness or strength of the culm wall and vascular bundle, and enhancing stem stability [121].

Another potential strategy to reduce plant height and improve lodging tolerance is the inhibition of plant growth regulators (PGRs) such as gibberellic acid (GA). GA inhibitors play a crucial role in the development of dwarf, semi-dwarf, and sturdier plants that can withstand lodging stress [110,122]. Semi-dwarfism and a reduction in plant height are caused by shorter internodes [123]. During the green revolution era, altered GA deficiency was considered the key in producing semi-dwarf rice and wheat cultivars with high yielding potential that boosted productivity of the two cereals [124]. Some major inhibitors of GA that have been utilized for the regulation of plant architecture include daminozide, mepiquat-Cl, chlormequat-Cl, and paclobutrazol (PBZ). PBZ (α-*tert*-Butyl-β-(4-chlorobenzyl)-1*H*-1,2,4-triazole-1-ethanol) inhibits GA precursor *ent*-kaurene conversion to *ent*-kaurenoic acid [125]. Some effects of PBZ include plant height reduction, enhanced nutrient uptake, and increased seed yield [126]. 

In millet crops, different approaches have been employed to mitigate or reduce lodging stress and improve yield. For example, in tef and finger millet, application of PBZ has been shown to reduce plant height and lodging stress [127]. Jency, et al. [128] reported that mutation in the kodo millet CO3 variety by gamma radiation or ethyl methane sulfonate (EMS) could produce nonlodging mutants. They also developed mutants (named second mutants or M2) which showed higher lodging tolerance due to photosynthetic efficiency (PhE) and culm thickness. Mutation of the alpha-tubulin-1 gene in tef by EMS produced the lodging-tolerant cultivar ‘Kegne’ [129]. 

**Table 1:** Effect of different abiotic stresses such as drought, heat, waterlogging, and lodging stresses on the yield of millet crops. Data represent percent yield loss in kg·ha^−1^ in response to a particular abiotic stress.

Stress	Finger Millet	Foxtail Millet	Wild Millet	Kodo Millet	Pearl Millet	tef	Little Millet	Proso Millet	References
Drought	61%	20.3%	30.1%		60.1%	69–77%	80.5%	64%	[28,62,130]
Waterlogging	42.14 kg/ha			42.84 kg/ha			18.14 kg/ha		[131]
Lodging		41.2% to 51.1%				30–35%			[109,132,133]
Heat	75% at 36/26 °C and 84% at 38/28 °C	60% at 38/28 °C			70 to 75% at 36/26 °C				[134,135,136]

## 3. Alternative Strategies for Enhancing Stress Resilience in Crops

### 3.1. Application of Plant Growth-Promoting Rhizobacteria (PGPRs)

The beneficial effect of PGPRs in improving abiotic stress tolerance and yield has been revealed in various crops [137,138]. PGPRs have been used to mitigate abiotic stresses and improve productivity in economically important crops including rice [139], soybean [140], lettuce [141], tomato [142], maize [143], and wheat [144,145,146]. One mechanism via which PGPRs improve plant performance is via the biosynthesis of essential plant growth regulators such as gibberellic acid (GA) and indole acetic acid (IAA). PGPRs were also found to trigger plant defense mechanisms and the biosynthesis of other growth regulators such as jasmonic acid and salicylic acid [147,148]. It was also found to help plants with nutrient acquisition from the soil in stress conditions [149].

#### 3.1.1. IAA Synthesis

Inoculation of IAA-synthesizing bacteria in various plant species resulted in enhanced root growth along with the formation of root hairs and lateral roots [150], resulting in improved nutrient and water uptake [151] and providing support to plants to tolerate osmotic stress [152]. Drought tolerance of plants increased manyfold due to IAA-synthesizing *Azospirillum* [150]. Production of hormones by bacteria and their activity in stimulating endogenous hormones contribute significantly to improving resistance [153]. Nitric oxide (NO) produced by *Azospirillum *brasilense** is involved in IAA signaling, which assists tomato (*Solanum lycopersicum*) plants in the formation of adventitious root [154]. Association of *A. brasilense* (strain Cd) to bean (*Phaseolus vulgaris* L.) during drought condition led to an enhancement in specific root length and root projection area as compared to control without *A. brasilense* inoculation [155]. 

#### 3.1.2. PGPR Effects on Root Morphology under Drought

Cell membranes play a great role in maintaining the physiological status of plant cells. Rhizobacteria influence processes that take place in the cell membrane. For example, in wheat seedlings, *Azospirillum brasilense* reduces the cell membrane potential, while, in cowpea, it was shown to decrease the phospholipid level of cell membranes and cause fluctuations in proton efflux [156]. Water deficit was reported to reduce phosphatidylethanolamine, alter root phospholipid composition, and improve phosphatidylcholine [157]; however, introduction of *Azospirillum* to wheat seedlings prevented these alterations, although lower phosphatidylethanolamine unsaturation and higher phosphatidylcholine were detected [158]. Alterations conferring elasticity in the cell membrane of roots due to bacterium-mediated changes is the primary mechanism for increased resistance to osmotic deficit [150]. The stability of cell membranes in plants is enhanced by the presence of PGPRs, which activate the antioxidant defense system, leading to increased resistance in plants against drought [159].

#### 3.1.3. Activity of ACC Deaminase-Synthesizing Rhizobacteria

Under stress conditions, endogenous ethylene maintains homoeostasis, leading to diminished shoot and root growth. Aminocyclopropane-1-carboxylic acid (ACC) is a precursor for ethylene biosynthesis [160], which is acted upon by bacterial ACC deaminase to impart energy and nitrogen to the plant [161]. In addition, the removal of ACC enables the bacteria to reduce ethylene toxicity, promoting growth and ameliorating stress [162]. *Achromobacter piechaudii* strain ARV8 which produces ACC deaminase was shown to improve the weights of pepper (*Capsicum annuum*) and tomato seedlings, as well as drop ethylene synthesis during saline stress [163]. Colonization of PGPRs from water-deficient areas due to tandem dry episodes are more stress-adapting and plant growth-promoting compared to bacteria colonized from sites where water is abundantly available [163]. Treatment of tomato seedlings with *A. piechaudii* ARV8 obtained from an arid site promoted growth as compared to seedlings treated with GR12-2 of *P. putida*, which was obtained from grass rhizosphere where water is abundant [164].

#### 3.1.4. Volatile Compounds and Drought Tolerance

Soil microbes when interacting with plant roots produce chemical compounds which are either organic volatile and inorganic volatile compounds in the form of gases which diffuse through the gaps in the soil particles or nonvolatile compounds (siderophores and phytohormones) [165]. These compounds play vital roles in the food chain of microbes, as well as in promoting plant growth, by improving plant biomass and defense systems against the plant pathogens through induced systemic resistance [166,167,168,169]. In plants, exposure to multiple stresses at the same time requires the functioning of volatiles [170,171]. These molecules introduced during stress conditions take part in signaling for generating systemic and priming effects within the same and nearby plants [172,173]. Enrichment with the AZP2 strain of *Bacillus thuringiensis* in wheat seedlings led to an increased biomass of plant and a five-fold increased survival rate during drought conditions because of a major decline in volatile emissions and photosynthesis enhancement [145]. These findings prove that introduction of bacteria contributes to stress resistance in plants [145]. These molecules stand as major participants for assessing drought and its mitigation through rapid, noninvasive techniques [145]. Growth of *P. chlororaphis* O6 in roots reduced loss of water by regulating stomata pores through a volatile metabolite known as 2*R*,3*R*-butanediol, whereas bacteria lacking such metabolite production did not show any sign of drought tolerance. This volatile also assists in introducing resistance in *Arabidopsis* during times of stress [174].

**Figure 1:**
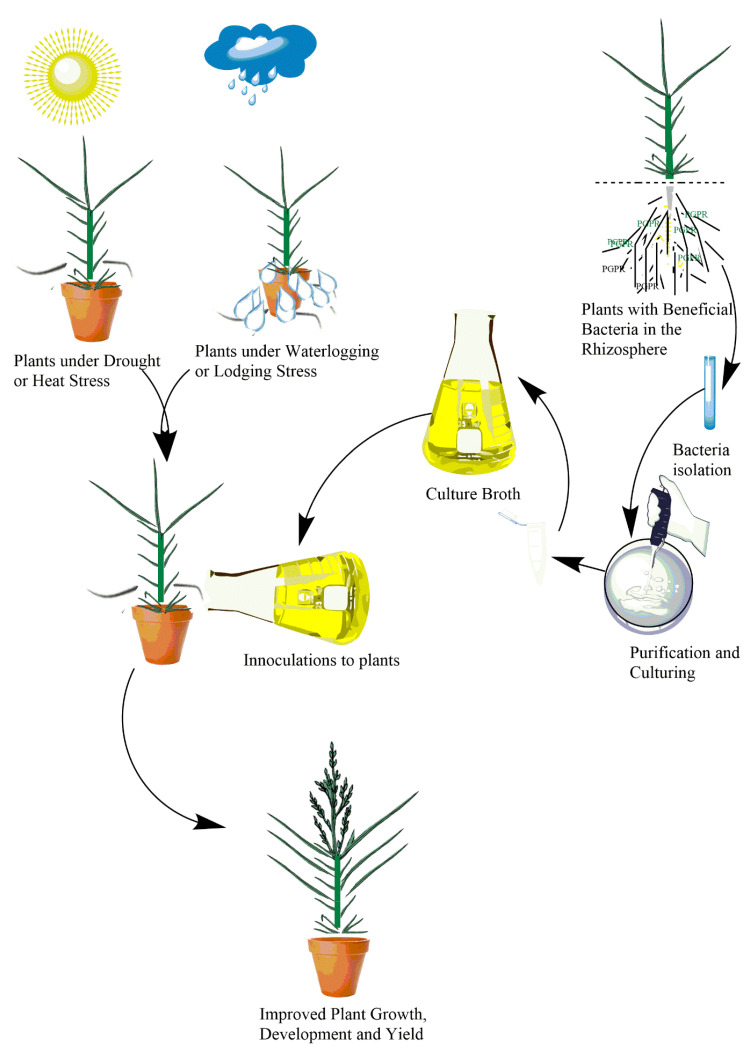
Potential application of PGPRs in abiotic stress mitigation and crop yield improvement in millets. PGPRs can be isolated from plants harboring the bacteria. The PGPRs are then cultured in a laboratory and applied to the soil of plant-growing substrate.

Given their application in improving plant performance under stress conditions in different crop plants as mentioned above, PGPRs have great potential to boost productivity of millets under abiotic stress conditions. A graphical illustration of the potential applications of PGPRs in millet crops is presented in Figure 1.

### 3.2. Application of CRISPR/Cas9 to Improve Stress Resilience in Millets

Site-specific nuclease (SSN)-based genome editing, developed in the last decade, has enabled effective and precise gene modification in plant and animal systems. The SSNs create double-strand breaks (DSBs) in their target DNA. These breaks are repaired via pathways such as homology-directed recombination (HDR) or nonhomologous end joining (NHEJ) that lead to mutations such as substitutions and insertion/deletions in the target regions [175]. Genome-editing techniques are used to produce mutants with defined phenotypes in contrast to the transgenic approach, in which the foreign DNA is randomly inserted into the genome and may or may not produce the desired phenotype [176]. Thus, the genome-editing technique is becoming a potent tool in crop breeding and functional genomics. Plants carrying edited genomes have the advantage of carrying modified DNA for a particular trait [177], while new varieties developed using this method can be used directly, unlike transgenic plants, with fewer concerns for consumers. Genome-edited plants carrying new alleles can also be used in breeding programs because of lower regulatory protocols as compared to genetically modified ones [178].

The CRISPR/Cas9 genome-editing technique has been used in more than 20 plant species [179] for improving various traits such as biotic and abiotic stress resilience and yield improvement [180,181]. Selection of a target gene is crucial to achieve the improvement of a desired trait. There are mainly two categories of genes that can be targeted for trait improvement: regulatory and structural genes. Proteins encoded by structural genes directly affect a trait, for example, abiotic stress tolerance [182], while regulatory genes act indirectly by controlling expressions of other genes that may also be involved in other cellular processes [183]. Furthermore, *cis*-regulatory sequences also play a crucial role in controlling abiotic stress tolerance [182]. In plants, the CRISPR/Cas9 system has been successfully employed in species such as cotton [184], maize [185,186], rice [187,188], and wheat [188,189] for abiotic stress mitigation. However, application of the CRISPR technique is limited to a few plant species, and it has focused on improvement of traits such as biotic stresses (diseases and insect pests), whereas its application for improving abiotic stress resilience and crop yield is limited. Recently, CRISPR/Cas9 was used to improve heat tolerance by targeting the *SlAGAMOUS-LIKE 6 (SIAGL6)* gene, which showed enhanced fruiting ability under heat stress in tomato [190]. CRISPR/Cas9 was also used for drought tolerance in maize by regulating the ARGOS gene without affecting the yield of the crop [181,191]. CRISPR/Cas9 technology has tremendous potential in developing multi-stress-resilient crops via simultaneous expression of many structural and regulatory genes in crop plants. Multiple gene editing via CRISPR/Cas9 has been performed for some crops including cotton [192], maize [185], wheat [193], and rice [187]. Single-base editing system of CRISPR-Cas has further widened its applications for the improvement of many important traits in crop plants [194,195]. Single-nucleotide changes in an essential domain of a gene may lead to loss-of-function mutation. It is thought that this technique may replace the traditional plant breeding approaches which were mostly based on the presence of populations with enough genetic variations for introducing desired traits to particular crop cultivars [196,197]. The base-editing technique of CRISPR/Cas9 can produce allelic variants in a particular population, thus leading to a desirable trait which can be identified by gRNA sequencing [198]. Thus, underutilized orphan crops such as millet could benefit from the immense potential of CRISPR/Cas9 genome-editing technology for environmental stress resilience and yield improvement. Table 2 summarizes the list of candidate genes which can be edited in millet crops through the CRISPR/Cas9 technique.

As incorporation of millet crops in food security programs is attracting increasing interest, the improvement of millets via the application of CRISPR/Cas9 and other gene-transformation technologies is being considered by the scientific community. For example, Mamidi, et al. [236] reported the genome assembly of *Setaria viridis* for the identification of important loci for traits such as loss of shattering and leaf angle, which are considered important yield predictors in many grass crops. They further validated the *Less Shattering1* (*SvLes1*) gene through CRISPR/Cas9 to control seed shattering. In other studies, *Agrobacterium*-mediated transformation was previously performed in foxtail millet (*Setaria italica*) for downregulation of phosphate transporters (SiPHT1;2, SiPHT1;3, and SiPHT1;4) [237]. They reported significant reductions in inorganic and total phosphate in root and shoot tissues, as well as an increase in the number of roots and hairy roots for the nonredundant roles. Furthermore, foxtail millet was the first millet crop to be sequenced [50]. The genome sequence of pearl millet has also been published [53]. Another study reported the successful production of transgenic finger millet (*Eleusine coracana* (L.) Gaertn.) plants through *Agrobacterium* [238]. The *Agrobacterium*-mediated transformation protocol has been developed for finger millet, and four cultivars of finger millet have been successfully regenerated through *Agrobacterium* transformation techniques [239]. Johnson, et al. [240] performed genome-wide population studies of three important millet crops: proso millet, little millet, and kodo millet. They identified various SNIPs: 3461 in kodo millet, 2245 in little millet, and 1882 for proso millet. These findings could help in genome editing for stress resilience and crop improvement.

**Figure 2:**
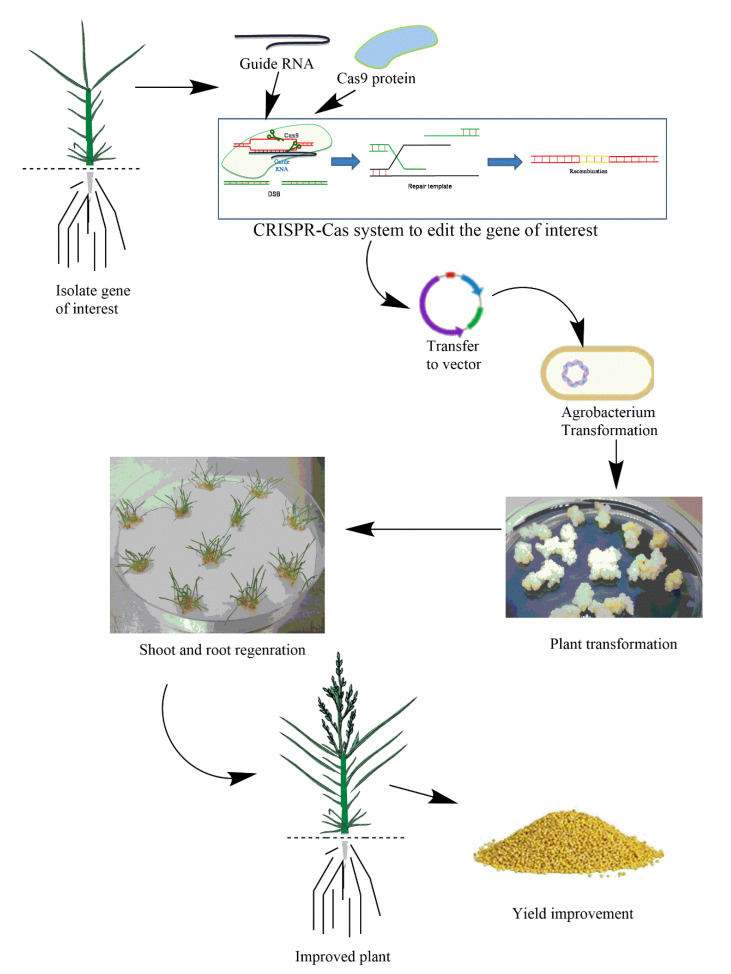
Illustration of CRISPR/Cas9 application in genome editing for yield improvement and stress mitigation. For CRISPR/Cas9 gene editing, a guide RNA (gRNA) is designed from a gene of interest, such as those listed in Table 2, and inserted into a binary vector. A bacterial CAS9 Nick-ase enzyme (Cas9) protein is also inserted into a binary vector. The gRNA and Cas9 expression cassettes are then used for *Agrobacterium*-mediate transformation for trait improvement. The fox-tail millet plant is used as a new model plant for CRISPR/Cas9 editing [241].

Recently, a foxtail millet mutant was generated by EMS-induced mutagenesis [241]. A point mutation named ‘Xiaomi’ in the light receptor gene phytochrome C (PHYC), which is essential in photoperiodic flowering and has rapid cycling time, was developed [242]. A CRISPR/Cas9 system that can be used to edit millet crops to improve stress resilience and yield is illustrated in Figure 2. We recently summarized tef homologs of major abiotic stress-responsive genes identified in related monocots such as rice, wheat, and maize, and we suggest those genes for CRISPR/Cas9 editing in tef for stress resilience and crop improvement [243].

## 4. Conclusions and Future Prospects

Millet crops have vital nutritional benefits as compared to other cereals. Nearly all millet crops have innate mechanisms to cope with certain environmental stresses such as heat, drought, lodging, and waterlogging, yet these stresses remain a threat to millet production with increasing impact of climate change. Techniques such as PGPRs and CRISPR/Cas9 are being used in other crops to lessen the impact of abiotic stresses, as well as to improve the productivity of crops. In this paper, we reviewed available literature on the subject matter and projected that the use of PGPRs and CRISPR/Cas9 will not only enable the plants to grow well in adverse conditions but also improve their yield significantly. Some candidate genes that can be targeted for manipulation by the CRISPR/Cas9 system to improve the growth and yield of millet crops have been suggested. Genomics, transcriptomics, metabolomics, proteomics, and other fields of study will also complement the alternative strategy we put forward. Improvement of millets has lagged behind that of major food crops and deserves increased attention from geneticists, biotechnologists, breeders, germplasm conservationists, etc. to improve global food security amidst climate change that is increasingly affecting the productivity of staple crops.

## Figures and Tables

**Table 2:** List of candidate genes from related species for potential CRISPR/Cas9 editing in millets.

Crop species	Candidate Genes for Editing in Millets	References
**Candidate genes for drought tolerance **		
*Oryza sativa*	*OsDIS1, OsiSAP7, OMTN2, OMTN3, OMTN4, OMTN6*	[199,200,201]
*Arabidopsis thaliana*	*ARR1, ARR10, ARR12, AtPUB19, FtMYB10, RGLG2*	[202,203,204,205]
*Nicotiana benthamiana*	*GhWRKY17*	[206]
*Triticum aestivum*	WRKY mRNA, TaWRKY146	[207,208]
*Eleusine coracana*	Threonine dehydratase mRNA	[21]
*Oryza sativa Japonica*	OsCDPK7, NF-Y18, Arginine decarboxylase (ADC), CIPK12	[209,210,211]
*Zea mays*	NF-YB	[212]
**Candidate genes for heat tolerance**		
*Triticum aestivum*	TamiR159, *TaHsfA6f, TaMBF1c, TaFER-5B, TaOEP16-2-5B, TaB2, TaGASR1*	[213,214,215,216,217,218,219]
*Oryza sativa*	hsp101, ZFP, OsWRKY11, OsGSK1, OsHsfA2e, mtHsp70, sHSP17.7, FAD7, SBPase, Sp17 (rice spotted leaf gene)	[220,221,222,223,224,225,226,227,228]
**Candidate genes for dwarfism (lodging tolerance)**		
*Oryza sativa Japonica*	KO_2_	[229]
*Zea mays*	GA regulatory factor-like (GRF) mRNA	[230]
*Oryza sativa Indica*	Growth-regulating factor 10 (GRF10)	[231]
*Oryza granulata*	GA20-oxidase (GA20ox2)	[232]
*Triticum aestivum*	BRI1, Rht1	[233,234]
*Oryza sativa*	Sd-1 (used in green revl)	[235]

## Data Availability

Not Relevant.
